# Computational Exploration of the Mechanism of Action of a Sorafenib-Containing Ruthenium Complex as an Anticancer Agent for Photoactivated Chemotherapy

**DOI:** 10.3390/molecules29184298

**Published:** 2024-09-11

**Authors:** Pierraffaele Barretta, Fortuna Ponte, Daniel Escudero, Gloria Mazzone

**Affiliations:** 1 Department of Chemistry and Chemical Technologies, University of Calabria, Via P. Bucci, 87036 Rende (CS), Italy; pierraffaele.barretta@unical.it; 2 Department of Chemistry, KU Leuven, Celestijnenlaan 200F, 3001 Heverlee, Belgium; daniel.escudero@kuleuven.be

**Keywords:** ruthenium complexes, sorafenib, PACT, PDT, photosensitizer, ISC

## Abstract

Ruthenium(II) polypyridyl complexes are being tested as potential anticancer agents in different therapies, which include conventional chemotherapy and light-activated approaches. A mechanistic study on a recently synthesized dual-action Ru(II) complex [Ru(bpy)_2_(sora)Cl]^+^ is described here. It is characterized by two mono-dentate leaving ligands, namely, chloride and sorafenib ligands, which make it possible to form a di-aquo complex able to bind DNA. At the same time, while the released sorafenib can induce ferroptosis, the complex is also able to act as a photosensitizer according to type II photodynamic therapy processes, thus generating one of the most harmful cytotoxic species, ^1^O_2_. In order to clarify the mechanism of action of the drug, computational strategies based on density functional theory are exploited. The photophysical properties of the complex, which include the absorption spectrum, the kinetics of ISC, and the character of all the excited states potentially involved in ^1^O_2_ generation, as well as the pathway providing the di-aquo complex, are fully explored. Interestingly, the outcomes show that light is needed to form the mono–aquo complex, after releasing both chloride and sorafenib ligands, while the second solvent molecule enters the coordination sphere of the metal once the system has come back to the ground-state potential energy surface. In order to simulate the interaction with canonical DNA, the di-aquo complex interaction with a guanine nucleobase as a model has also been studied. The whole study aims to elucidate the intricate details of the photodissociation process, which could help with designing tailored metal complexes as potential anticancer agents.

## 1. Introduction

Cancer and cardiovascular diseases continue to be the main causes of death worldwide [1]. With the aim of overcoming the well-known limitations associated with current cancer treatments and increasing the range of treatable cancers, novel strategies have been proposed in recent years. Today, the accessibility of highly focused light sources has established photoactivation approaches, such as photodynamic therapy (PDT) and photoactivated chemotherapy (PACT), as alternative and emerging treatments against cancer. Both techniques provide a therapeutic agent that can be activated by specific light wavelengths; however, they differ in terms of the mechanism of action of the drug in reaching a specific target. PDT involves the use of a photosensitizer (PS) that accumulates selectively in cancerous tissues and generates reactive oxygen species (ROS), highly reactive agents, which finally induce oxidative damage to cellular components [2,3,4,5,6]. The photodynamic action means that once a photon is absorbed by the PS, one of its excited singlet states is populated. Thereafter, intersystem crossing (ISC) processes populate triplet excited states, which, if they have suitable characteristics, can transfer their energy or electrons to molecular oxygen, thus producing ROS. This behavior makes PDT an oxygen-dependent therapy and limits its use in a hypoxia environment. In contrast, PACT is an innovative approach that integrates the principles of chemotherapy with PDT techniques, demonstrating efficacy even in hypoxic cancer cells [7,8]. An ideal candidate for PACT would be a prodrug that exhibits high stability and non-toxicity to cells in the dark. After light irradiation, particularly within tumor tissues, the prodrug should be activated, ensuring targeted therapeutic action, thus minimizing the damage to healthy cells. These compounds exploit various mechanisms to induce cell death [9,10], including photo-induced ligand ejection with the subsequent formation of the corresponding photoproduct and generation of ROS, similar to the mechanism of a traditional PDT agent.

Transition metal-containing complexes are considered highly promising systems for application in PACT due to their rich photophysical and photochemical properties, which can be finely tuned by varying the metal center and/or surrounding ligands [11,12,13,14,15]. In this context, ruthenium(II) complexes have appeared as a prominent class of compound among potential metal-based drugs [7,8,9]. For most Ru(II)-based PACT compounds, the accessibility to photoactive metal-centered (^3^MC) triplet states, which are characterized by their dissociative nature, is key to achieving efficient photochemical reactivity. The ^3^MC states play a pivotal role in influencing processes such as electron transfer and ligand dissociation and arise from triplet excited states of a metal-to-ligand character (^3^MLCT), which are themselves produced through rapid ISC processes from the manifold ^1^MLCT states.

**Scheme 1:**
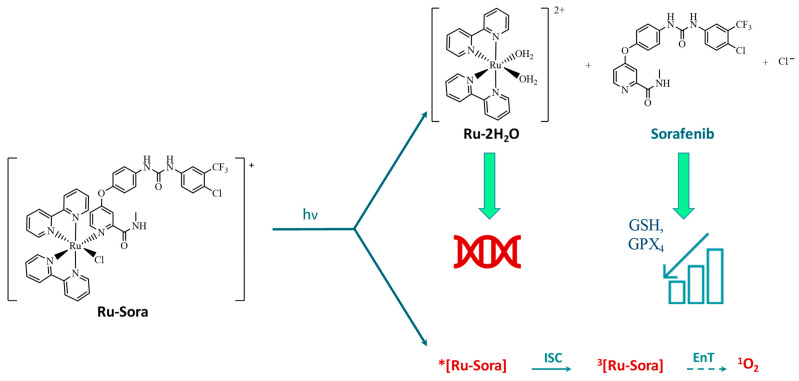
Schematic mechanism of action of the **Ru-Sora** complex, where ISC stands for intersystem crossing and EnT for energy transfer.

In the last years, research has been focused on the octahedral ruthenium(II) polypyridyl complex, a promising family of uncaging molecules [8,16]. These complexes can undergo photosubstitution reactions, leading to the formation of aquated species upon ligand dissociation, which in turn results in observed photocytotoxicity. The phototoxic ruthenium aquated photoproducts, directly interacting with DNA nucleobases, act as replication and transcription inhibitors as their binding to nuclear DNA entails a distortion of its structure. Recently, an attractive strategy has been to design Ru-based prodrugs with bioactive ligands, providing the advantage of a single prodrug performing multiple actions and targeting different cellular sites. Numerous examples can be found in recent literature, and their number is constantly increasing [9,17,18,19,20]. Recently, a novel photoactive Ru(II) complex has been synthetized, utilizing sorafenib, an antineoplastic agent, as a photoreleasable ligand [21]. This complex, [Ru(bpy)_2_(sora)Cl]^+^, where bpy = 2,2′-bipyridine and sora = 4-[4-[[4-cloro-3-(trifluorometil)fenil]carbamoilammino]phenossi]-N-metil-pyridine-2-carbossamide and named **Ru-Sora**, integrates photoactivated chemotherapy (PACT) based on metal with the sorafenib agent, which induces ferroptosis and can effectively eradicate hepatocellular carcinoma (HCC) cells. Experimental data have demonstrated that after light irradiation at 465 nm, **Ru-Sora** releases sorafenib, which indirectly interferes in the biosynthesis of glutathione (GSH), inhibits the expression of glutathione peroxidase 4 (GPX4), forms a ruthenium aquated complex (Ru-w) that can bind DNA, and generates singlet oxygen (^1^O_2_) that can induce apoptosis through both the extrinsic and intrinsic pathways [22] (Scheme 1). **Ru-Sora** has shown significant photoactive activity against Hep-G2 cells, including sorafenib-resistant variants. Its photocytotoxicity has been found to be superior to the effects of either sorafenib or ruthenium complexes alone. Thus, the mechanism of induced cell death is a combination of ferroptosis and apoptosis.

Motivated by the observed effectiveness of this recently synthesized PACT agent, we conducted a comprehensive computational investigation of the mechanistic aspects of **Ru-Sora** in order to shed light on its whole action. For this purpose, density functional theory (DFT) and the time-dependent approach (TDDFT) in its Tamm–Dancoff approximation (TDA) were used to explore the photophysical properties and the photodissociation mechanism of sorafenib. The electronic transitions accounting for the whole absorption spectrum and the associated bands were fully characterized. In order to consider the possibility of triplet-state population, pivotal for both PDT and PACT activities, the rate constants of ISC for the most probable deactivation channels of the singlet state were computed. The excited states, both singlet and triplet states, potentially involved in the photorelease of ligands were fully described. Additionally, to simulate the DNA attack that ultimately prevents replication and transcription, the interaction of the aquated complex with the purine guanine (Gua) nucleobase was also investigated. This study aims to contribute to designing efficacious Ru complexes suitable for PACT application by clarifying the key steps required for ligand release to occur.

## 2. Results and Discussion

### 2.1. Structural and Electronic Properties

**Figure 1:**
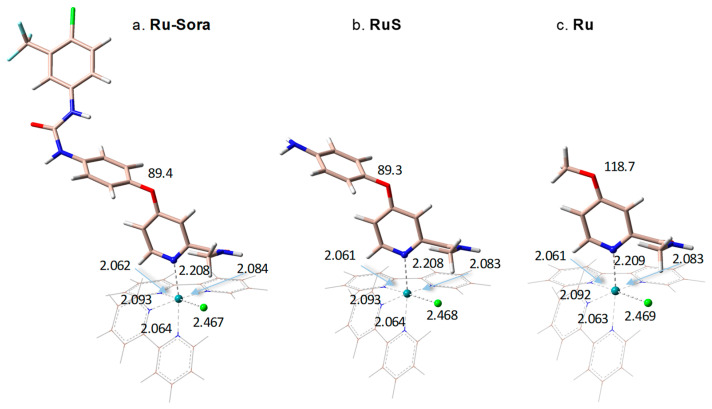
Optimized structures of the (**a**) synthesized **Ru-Sora** complex and the models (**b**) **RuS** and (**c**) **Ru**. Selected bond lengths and valence angles are reported in Å and degrees, respectively.

The optimized structures of all the investigated complexes are shown in Figure 1, where some key structural parameters are included. The synthesized **Ru-Sora** complex (Figure 1a) is characterized by the typical octahedral geometry adopted by Ru(II)-based polypyridyl complexes, [21] in which the longest coordination bond has been found for the chlorido ligand (2.467 Å). The two bpy ligands, instead, lie at a medium distance of 2.076 Å. The ligand that according to the proposed mechanism photodissociates, i.e., sora, displays a bond distance of 2.208 Å from the metal center.

In order to retain the computational effort, a preliminary study was carried out exploring the structural and electronic properties of the reference complex **Ru-Sora** and two model systems named **RuS** and **Ru** (see Figure 1b and Figure 1c, respectively). In the two models, the structure of sora was cut down either at the diamide group (-HN-C(=O)-NH-), which was replaced by the amine one (-NH_2_), or at the phenyl ether oxygen, which was replaced by the methoxy one (-OCH_3_), respectively. The comparison between the structural parameters included in Figure 1 clearly evidences that the model of sora does not influence the structural arrangement of the whole complex, i.e., the corresponding Ru–N bond distance remains around 2.208 Å for all the models.

**Figure 2:**
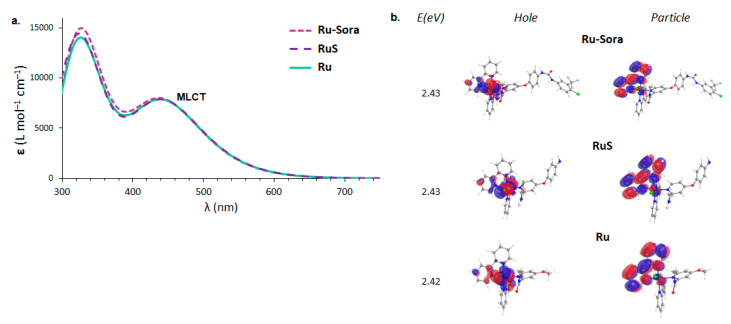
(**a**) Absorption spectra of the synthesized complex **Ru-Sora** [21] and the models **RuS** and **Ru**. (**b**) NTOs of the lowest-lying singlet state (λ_max_).

To ascertain that upon irradiation the behavior of the complex remains unchanged regardless of the model, the absorption spectrum of the **Ru-Sora**, **RuS,** and **Ru** complexes were also simulated. For TDA–DFT calculations, a preliminary benchmark on the reference complex **Ru-Sora** was carried out in order to properly choose the exchange and correlation functional, by comparing the wavelength for the maximum absorption (λ_max_), one of the key parameters in PDT. The outcomes are collected in Table S1, from which PBE0 emerged as one of the best choices looking at both the maximum absorption wavelength and the relative intensity of the two bands experimentally recorded [21], in accordance with previous reports on similar complexes [23,24,25,26,27,28,29]. In Figure 2, the absorption spectra of the three complexes, **Ru-Sora** and the models **RuS** and **Ru**, together with the natural transition orbitals (NTOs) associated with the λ_max_ are reported. In Table S2, the detailed TDA–DFT results for the three complexes are summarized.

The computed UV-Vis absorption spectra of **Ru-Sora**, **RuS,** and **Ru** almost completely superimpose each other. The comparison of their excitation energies as well as their NTOs plots confirms the same excited state character for the investigated complexes. From the analysis of the NTOs, it appears clear that in none of the cases does the charge transfer involve the 4-chloro-3-trifluoromethylphenylammide ring (missing in the **RuS** structure) or the 1-(4-chloro-3-(trifluoromethyl)phenyl)-3-phenylurea group (missing in the **Ru** structure). Therefore, it should not strongly impact the modeling of the photophysical properties of the complex. Therefore, the model **RuS** has been taken into consideration for further photophysical investigations. The **Ru** model, instead, was used for the complete characterization of the release ligand mechanism, discussed in the last section, the exploration of which required many attempts before finding the most probable mechanism.

**Figure 3:**
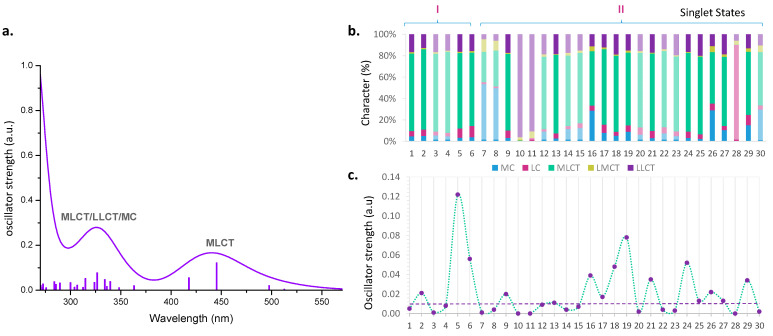
(**a**) Absorption spectra of **RuS** complex in which the main vertical electronic transitions (those with an oscillator strength (a.u.) greater than 0.01), together with the main character of the two bands, are reported; (**b**) percentage of metal-centered (MC), ligand-centered (LC), metal-to-ligand (MLCT), ligand-to-metal (LMCT) and ligand-to-ligand (LLCT) charge transfers character for each vertical transition; (**c**) oscillator strength (a.u.) for the first thirty electronic transitions.

The main TDA–DFT results for the selected model complex **RuS** are included in Table S2. In Figure 3, the computed absorption spectrum is reported, including the main vertical electronic transitions (those with an oscillator strength greater than 0.01 are shown as vertical bars), together with the main character of the excited states contributing to the two bands. In accordance with experimental records, in the region 300–600 nm, the complex exhibits two main bands with a similar intensity. The maximum absorption wavelength is located at 511 nm, though the computed oscillator strength is only 0.004. The associated electronic transition is HOMO(H)→LUMO(L) amounting to up to 70%. The band, instead, is centered at around 440 nm.

The character of each transition and, then, that of the two bands, was assigned using the fragment-based analysis approach, as implemented in the Theodore 3 package [30], where the system is divided into five fragments: the metal center, and the four ligands. The outcomes of the decomposition analysis are reported in Figure 3b. For the characterization of the bands, only those electronic transitions with a considerable oscillator strength (greater than 0.01 a.u.) have been taken into consideration (see Figure 3c), with the exception of the electronic transition associated with the S_1_ state, useful for the photodynamic action, which has also been included. Therefore, analyzing the selected electronic transitions belonging to the first band, what emerges is essentially an MLCT character, though a contribution of LLCT for ca. 20% can be observed in all the electronic transitions. Indeed, the most intense transition (445 nm) is originated by H-2→L (40%) and H-1→L (32%) electronic transitions, where the starting orbital in both cases is shared between the metal and a bpy or sora ligands, while the recipient of the transferred charge (L) involves both the bpy ligands. The higher-energy band, instead, is originated by several excitations leading to states of a different character. In the resulting band, indeed, though the MLCT contribution remains quite prominent (between 51 and 74%), we can also note LLCT (11–20%) and MC (3–29%) characters.

### 2.2. Photodynamic Processes

The photodynamic action starts with the radiationless transitions, i.e., ISC processes, from the singlet to the triplet manifolds, as the triplet state is the species responsible for promoting the excitation of molecular oxygen. Therefore, once the one-photon absorption occurs and a singlet state is populated, all the plausible deactivation channels of the singlet state that can lead to a triplet-state population should be analyzed. As stated above, the state that will most probably be populated upon irradiation is a higher-energy singlet state than the lowest-lying one. Nevertheless, the most accepted hypothesis provides the relaxation, through internal conversion, of higher excited states to the lowest one before ISC processes can occur [31,32,33]. 

**Figure 4:**
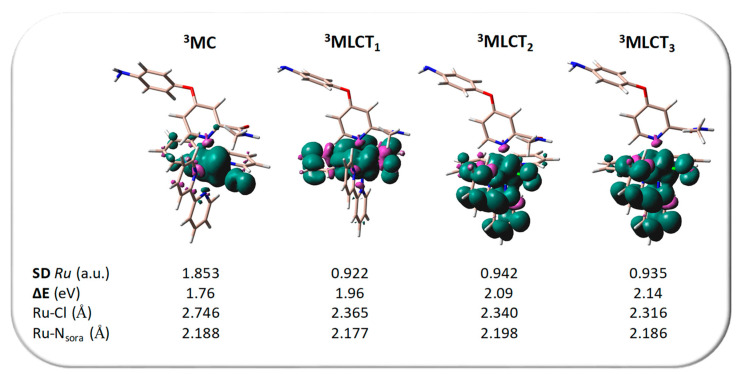
Computed spin density isosurfaces (accomplished with an isovalue of 1 × 10^−3^ a.u., envy and purple colors stand for positive and negative parts) of the intercepted TDA–PBE0 triplet states. The spin density of the metal center and adiabatic energy gap ΔE with respect to the GS are also provided.

In order to choose the number of triplet states to be considered for identifying the most probable deactivation channel of the singlet state, a primary analysis of the triplet states obtained from the vertical approximation was carried out. In Figure S1, the outcomes of the decomposition analysis conducted for the first eight triplet states are reported: six triplet states show a considerable MLCT character, while two of them present a great contribution of MC character. Noteworthily, the latter states should be those involved in the photodissociation processes. Therefore, in order to account for plausible ISC processes, the structure of the eight triplet states was fully relaxed, together with the structure of the singlet excited state potentially involved in the PDT action, S_1_. The optimization of the eight triplet states converged into four states, all located adiabatically below the energy of S_1_. To confirm the character of the resulting triplet states, the plot of spin density is reported in Figure 4.

For the triplet states located between 1.96 and 2.14 eV, a spin-density value on the metal center close to unity has been found, suggesting a MLCT character for all of them. On the contrary, the ruthenium spin density in the case of the triplet lying at 1.76 eV is 1.853 a.u., indicating the two unpaired electrons localized on the metal center. Interestingly, differently from what was expected, the lowest-lying triplet state is dissociative towards the chlorido release rather than sora ligand. Indeed, the Ru-Cl distance in ^3^MC (2.746 Å) is considerably longer than that of the other three triplet states (2.316–2.365 Å). On the contrary, the distance between the metal and the nitrogen atom of the sora ligand remains essentially invariant for all the optimized triplet excited states, varying not more than 0.023 Å with respect to the ground-state structure.

**Figure 5:**
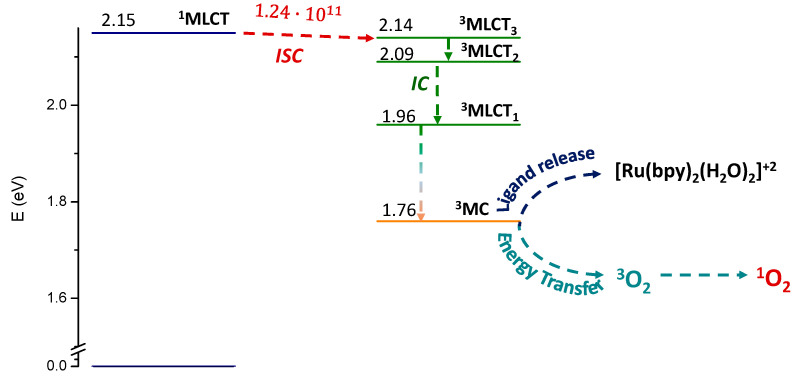
Jablonski-like diagram representing the mechanism of action of the **RuS** complex. The kinetic constant of the most probable ISC process starting from the lowest-lying singlet state (S_1_) is reported above the dashed red arrow in s^−1^.

Therefore, the photophysical behavior suggests that once the low-lying triplet state is populated, the photodissociation should primarily involve the chlorido ligand. Once the triplet states had been characterized, the rate for their population, starting from the lowest-lying singlet state, was computed as well. These values are collected in Table S3 and those involved in the most probable ISC process are included in Figure 5, where the adiabatic energies of the lowest-lying singlet and triplet excited states are reported.

The relaxation of the S_1_ state entails a stabilization of 0.27 eV; it is characterized by a slight shortening of the coordination Ru–N bonds, nevertheless the character remains of the type MLCT. Therefore, at this point, the ISC between the singlet and the triplet closest in energy and characterized by the same nature, ^3^MLCT_3_, could occur. The vanishing energy gap (0.01 eV) and a SOC value equal to 278.5 cm^−1^, accompanied by a computed k_isc_ of 1.24 × 10^11^ s^−1^, surely ensure the population of such a ^3^MLCT state. From here, fast internal conversion should entail the population of lower-lying triple states with an MLCT character, while, as will be discussed in the next section, the overcoming of a very low activation barrier is needed to yield the ^3^MC state.

The triplet-state energy higher than the threshold value of 0.90 eV, the energy required to excite molecular oxygen from ^3^$\Sigma_{g}^{-}$ to ^1^Δ_g_, is evidence of plausible ^1^O_2_ generation. Based on the experimental findings, the only ROS produced by **Ru-Sora** upon irradiation is ^1^O_2_. Nevertheless, computational strategies were exploited to explain the reason for this behavior, taking **RuS** as the model complex. According to previous reports about the ability of metal-based complexes to promote type I photoprocesses, the occurrence of such processes requires that the photosensitizer is able to lose and acquire electrons either in its ground or excited triplet state, in order to form radicals. Therefore, the vertical electron affinity (VEA) and ionization potential (VIP) of the PS in its ground and triplet excited states, generally indicated as [Ru]^+^ and ^3^[Ru]^+^, respectively, were determined for the **RuS** complex, and collected in Table 1, together with the VEA of molecular oxygen computed at the same level of theory.

The main one-electron transfer mechanisms associated with the type I pathway can be summarized as follows:

^3^[Ru]^+^ + [Ru]^+^ → [Ru]^2+●^ + [Ru]^●^ (1)

^3^[Ru]^+^ + ^3^[Ru]^+^ → [Ru]^2+●^ + [Ru]^●^ (2)

[Ru]^●^ + ^3^O_2_ → [Ru]^+^ + O_2_^−●^ (3)

Such photoprocesses usually begin with an initial one-electron reduction of the triplet-state PS (^3^[Ru]^+^) with the production of a PS radical ([Ru]^●^) (reaction 1). Alternatively, a one-electron transfer can occur between two neighboring molecules of the PS in its triplet state (^3^[Ru]^+^) (reaction 2). The PS radical ([Ru]^●^) produced can further transfer one electron to molecular oxygen to produce the superoxide radical O_2_^•−^ (reaction 3), which, in turn, can be reduced to hydrogen peroxide H_2_O_2_ by, for example, superoxide dismutase (SOD).

The occurrence of autoionization reactions 1 and 2 can be established by looking at the sum of the ground-state VIP and triplet-state VEA (reaction 1), and at the sum of the VEA and VIP of the triplet state (reaction 2), which should be less than zero for the reactions to be energetically favored. While for the first reaction the sum leads to a positive value (0.65 eV), in the second case the result is negative (−1.39 eV), suggesting that the triplet state of the complex, contrariwise to the ground one, can effectively produce a complex radical ([Ru]^●^) that, in principle, can further react with molecular oxygen according to reaction 3. Nevertheless, the latter reaction is energetically disfavored, as the VEA of [Ru]^+^ is higher than that of molecular oxygen, leading to an energy reaction of 0.4 eV.

Alternatively, the PS in its triplet state can directly interact with molecular oxygen to achieve superoxide anion according to the following reaction:

^3^[RuS]^+^ + ^3^O_2_ → [RuS]^2+●^ + O_2_^−●^ (4)

However, it is difficult for the ionization of the PS triplet state to occur, its VIP being 3.13 eV, which is significantly higher than the absolute value of molecular oxygen VEA. Therefore, though the production of the PS radical [Ru]^●^ can be achieved according the viability of reaction 1, it cannot further react to produce the ROS O_2_^–●^ essential for the production of H_2_O_2_, which can be transformed into highly oxidative OH^•^ by reacting with O_2_^•−^ or Fe^2+^ (Haber–Weiss and Fenton reactions, respectively) [34].

### 2.3. Sorafenib Release Mechanism

**Figure 6:**
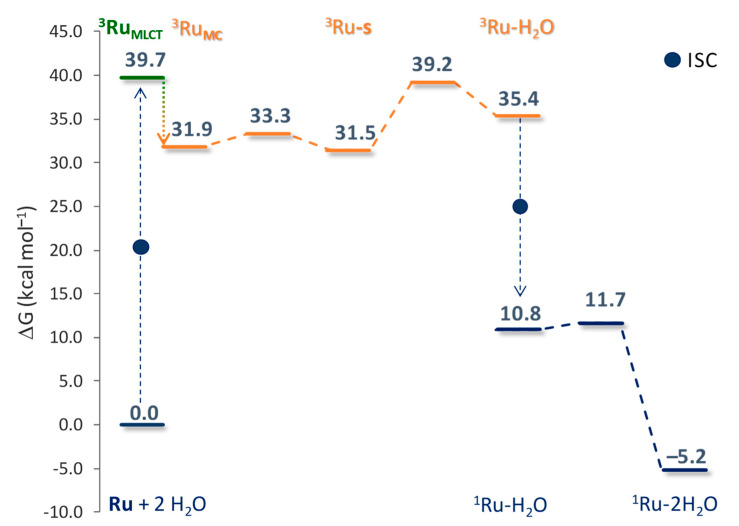
Calculated B3LYP-D3 free energy profile describing the activation mechanisms of **Ru** complex. Energies are in kcal mol^−1^ and relative to separated reactants, which are **Ru** complex and two water molecules.

Upon exposure to visible light, ruthenium complexes can undergo a photochemical substitution reaction, resulting in the replacement of ligands within the coordination sphere of the metal center by solvent molecules. Computational strategies have proven indispensable to study in depth the photoactivation mechanisms of several Ru(II)-based complexes, elucidating light-induced photodecomposition pathways of excited states and monitoring the photochemical reactions [8,35,36,37,38,39,40]. The whole reaction along the free energy profile of the excited triplet state, taking **Ru** as the model complex, is reported in Figure 6. All the stationary points, both minima and transition states, have been identified and individually characterized. Relative free energies in solution have been calculated with respect to the ground-state singlet reactants, which are the **Ru** complex and two water molecules (**Ru** + 2H_2_O).

The first steps described above show that **Ru** complex, when irradiated by light of the appropriate wavelength, gives rise to the metal-to-ligand charge transfer (^1^MLCT) state. An ISC process, thus, populates the triplet state, indicated here as ^3^Ru_MLCT_. These steps are summarized in the figure with the dashed blue line from the ground state to the ^3^Ru_MLCT_ state. The latter is located 39.7 kcal mol^−1^ above the ground state. As already stated above for the complex alone, such a triplet state with the surrounding water molecules exhibits only minimal geometric structural differences compared to the ground state. The deactivation pathway of the ^3^Ru_MLCT_ state involves the population of the nearby triplet MC state, here named ^3^Ru_MC_. To estimate the energy required to pass from one to the other triplet state, a relaxed scan was performed. The corresponding potential energy surface is shown in Figure S2 of the Supplementary Materials as obtained by elongating the Ru–Cl distance by a step of 0.01 Å. An abrupt transition from the ^3^Ru_MLCT_ state to the ^3^Ru_MC_ one is observed at the 2.56 Å distance, for an estimated barrier of only 1.5 kcal mol^−1^. This state, characterized by a dissociative nature, lies 7.8 kcal mol^−1^ below the previous one. As can be seen from the spin-density plots included in Figure S2, the two triplet states involved in the conversion are the lowest-lying triplet states ^3^MLCT_1_ and ^3^MC discussed above. Indeed, the key feature of the ^3^Ru_MC_ state, already found for the complex alone, is the significant elongation of the Ru-Cl bond, which increases by 0.52 Å compared to the ground state. This bond elongation, indicative of the dissociative character of the triplet ^3^Ru_MC_ state, facilitates the hydrolysis process of the complex that proceeds via a gradual mechanism.

The complete dissociation of the chlorido ligand occurs through a dissociative mechanism, leaving a vacant coordination site on the ruthenium center, which results in the formation of ^3^Ru-s, a five-coordinated species. The barrier for the corresponding transition state is only 1.4 kcal mol^−1^ and the imaginary frequency is 111.9*i* cm^−1^. In the subsequent step, a water molecule enters the coordination sphere of the metal and the simultaneous release of the sora ligand occurs. This process leads to the formation of the penta-coordinated monoaquo Ru complex indicated as ^3^Ru-H_2_O. The formation of this monoaquo complex requires overcoming an energy barrier of 7.7 kcal mol^−1^. The concerted associated transition state has an imaginary frequency of 141.4*i* cm^−1^. Several investigations into the formation of the final diaquo product along the triplet state surface have been carried out, but all the attempts failed. This evidence suggests that the formation of the final product, Ru-2H_2_O, could occur via the ground singlet multiplicity. Specifically, the triplet adduct undergoes an intersystem crossing to the corresponding singlet adduct, which lies 10.8 kcal mol^−1^ above the zero reference. Along the singlet path, a second water molecule approaches the penta-coordinated ^1^Ru-H_2_O complex in an associative manner, completing the hydrolysis process and overcoming a very low activation energy barrier of only 0.9 kcal mol^−1^. The imaginary frequency that confirms the nature of this stationary point is 28.3*i* cm^−1^. The final product formation is calculated to be exothermic by 5.2 kcal mol^−1^. The optimized structures of all the stationary points intercepted along the pathway just described are collected in Figure S3.

**Scheme 2:**
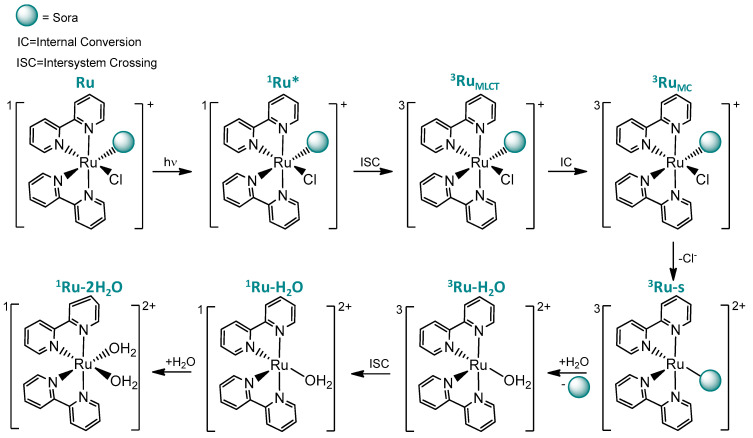
Proposed activation mechanism of **Ru-Sora** complex.

Summarizing the outcomes, Scheme 2 illustrates the light-induced activation of the **Ru-Sora** complex. When the complex is irradiated by light with the appropriate wavelength, this gives rise to ^1^MLCT state, denoted in the scheme as ^1^Ru*. After, through a radiationless ISC, from the ^1^Ru* state to the ^3^MLCT_3_ state followed by internal conversion to ^3^MLCT_1_, the ^3^Ru_MLCT_ state is generated. Then, the overcoming of a very low energy barrier leads to the formation of the lowest-lying triplet-state ^3^Ru_MC_, the pivotal excited state in the photoactivation reaction. From here, the removal of the chloride from the coordination sphere of the metal is accompanied by the formation of a penta-coordinated complex, in which the arrangement of the other ligands around the metal remains unchanged, therefore leaving vacant one coordination site of the metal.

The entrance of the water, instead, is accompanied by the release of the sora ligand, thus maintaining the penta-coordinated nature of the complex ^3^Ru-H_2_O. From here, the complex needs to decay to the ground-state ^1^Ru-H_2_O in order for the second water molecule entrance to occur. The formation of the final product, which is the diaquo complex ^1^Ru-2H_2_O, is accomplished by overcoming a very low energy barrier. The slowest step, instead, is the second one, involving the entrance of the first water molecule in the place of the sora ligand, which requires 7.7 kcal mol^–1^ to take place. It is worthy of note that the entire reaction, with the involvement of the triplet state ^3^MC, occurs with a rather low energy cost.

### 2.4. Interaction of Ru-2H_2_O with DNA

Although the final action of this type of Ru(II)-based complex involves the interaction of the aquated complex with DNA [28,41,42,43,44], only recently has an in-depth computational study been performed on this step of the anticancer action. The examined complex is characterized by the presence around the ruthenium center of two N^N chelate ligands, bpy and bathocuproine, apart from the two water molecules, similarly to the diaquo complex studied here, which presents two bpy ligands [45].

As is well known, under the action of conventional Pt(II)-based anticancer drugs, DNA platination occurs by following a one-step associative mechanism in which the N7 guanine coordinates the metal in the place of a water molecule, similarly to the aquation step widely described in the literature for a series of Pt(II)-based complexes [46,47,48,49]. On the contrary, as already described above for the aquation steps, the ruthenium center induces a dissociative mechanism for the ligand substitution reactions. Indeed, in most of the steps analyzed above, the exit of a ligand and the entrance of the incoming ligand involves the formation of a penta-coordinated complex in which a coordination site remains vacant, without changing the arrangement of the remaining ligands around the metal center.

**Figure 7:**
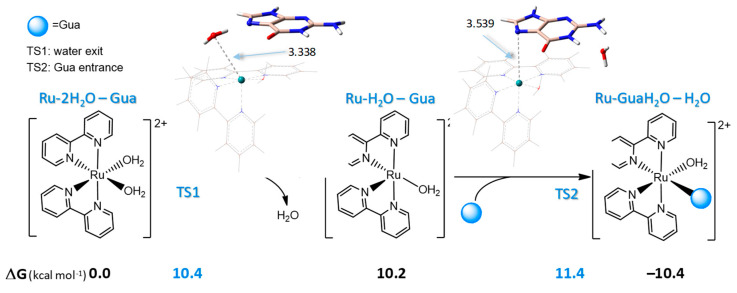
Reaction mechanism found for the substitution reaction of a water molecule with the guanine purine base. Relative Gibbs free energies (kcal mol^−1^) are reported in bold. The optimized structure of the intercepted transition states is reported above the arrows. For the sake of clarity in the sketched minima structures, the species around the metal complex, either guanine or water, are omitted.

The model widely used to account for DNA platination entails the simulation of a DNA attack by the metal center with the substitution reaction of a water molecule with the purine guanine nucleobase, recognized as the most probable DNA site attack for the widely used Pt(II)-based complexes [50]. Therefore, the attack of Ru-2H_2_O to DNA has been simulated by considering the attack of N7-guanine to the Ru(II) center, causing the displacement of a water ligand. The outcomes of this exploration are reported in Figure 7, where the energies put into play and the transition states intercepted along the pathway can be seen.

The reaction starts with the formation of an initial adduct between the reacting species, chosen as a reference (ΔG = 0.0 kcal mol^−1^), and here reported as Ru-2H_2_O – Gua. In this adduct, hydrogen bonds between both the N7 guanine and an H atom of the leaving water molecule and the oxygen of guanine with an H atom of the other water ligand are established. From here, only a stepwise mechanism has been located for the entrance of the nucleobase in the coordination sphere of the metal in place of a water ligand. The first step of the reaction involves the elongation of the Ru–water coordination bond up to 3.338 Å and requires 12.3 kcal mol^−1^ to occur. The movement associated with the located transition state TS1, with an imaginary frequency of 169*i* cm^−1^, evidences only the exit of the water ligand, which leads to the penta-coordinated complex Ru-H_2_O that lies 10.3 kcal mol^−1^ above the reference. In this complex, the metal center presents a vacant coordination site, similar to the complex found in the last step of the sora release, ^1^Ru-H_2_O. The IRC calculations confirm the proper connection, through TS1, between the esa Ru-2H_2_O complex and the penta Ru-H_2_O. The second step starts from the just formed penta complex and involves the approaching of guanine to the metal center. The transition state accounting for this movement was located at 1.2 kcal mol^−1^ from the preceding minimum; this clearly shows the involvement of the sole nucleobase in the associated imaginary vibrational frequency (27*i* cm^−1^), which lies 3.539 Å from the metal center. The formation of the final product, in which the nucleobase is definitively coordinated to the metal center (Ru-N_Gua_: 2.141 Å), is exergonic by 10.4 kcal mol^−1^.

Therefore, in the interaction of Ru-2H_2_O with DNA, the slowest step involves a water ligand leaving the metal coordination sphere, similarly to the complex bearing a batocuproine ligand in the place of a bpy one [45]. With respect to this complex, a slightly higher energy cost is required for such a step to occur. However, comparing the energy put into play for the displacement of a water ligand in favor of the guanine in the investigated Ru(II)-based complex with those reported in the literature [46,49,50], a good proclivity of the diqauo Ru(II) complex, Ru-2H_2_O, to attack the DNA can be underlined as no more than 10.4 kcal mol^−1^ is required for the reaction to take place.

## 3. Methods

The geometries were optimized within the DFT framework using the Gaussian 16 package [51]. In order to explore the main properties of the synthesized complex **Ru-Sora** and the designed models **RuS** and **Ru**, the hybrid functional B3LYP [52,53], including the Grimme’s empirical dispersion (GD3), was used and coupled with the standard 6-31G(d) basis set, which was used for all the atoms, except the Ru center for which the mwb28 effective core potential and the corresponding valence basis set [54] were employed [35,36,55]. All the calculations were performed in a water environment (ε = 78) by using the CPCM polarizable conductor calculation model [56]. A preliminary benchmark (Table S1 of the Supplementary Materials) on the maximum absorption wavelength of **Ru-Sora** complex was carried out in order to select the most suitable exchange and correlation functional to be used for the simulation of the absorption spectrum of the investigated complexes. The time-dependent approach (TDDFT) in its Tamm–Dancoff approximation (TDA) [57] was employed to optimize the structures of the excited states selected for the PDT action. Among B3LYP [52,53], CAM-B3LYP [58], B3PW91 [52,59], M06 [60], M06L [61], PBE [59], PBE0 [62], LC-WHPBE [63], ωB97 [64], ωB97X [64], B97D [65], and ωB97XD [64], PBE0 emerged as one of the most suitable functionals to use for further studies. To correctly characterize the electronic transitions, the fragment-based analysis was computed with TheoDORE 3 (Theoretical Density, Orbital Relaxation and Exciton analysis) software [30].

The ORCA 5.0.4 package [66] was used to calculate the ISC rate constants between the singlet and triplet excited states lying below by using the equilibrium geometry of each triplet state, employing the ESD-TDDFT approach as performed in our previous work [5,67]. Hessian matrices of the involved excited states were computed on the TDA-PBE0 optimized structures of each geometry. The rate constants obtained are the average of the contributions of the three spin substates (MS) = {+1, 0, −1}. Relativistic corrections were computed by the zeroth order regular approximation (ZORA) employing the B3LYP functional. ZORA-DEF2-TZVP and old-ZORA-SVP basis sets were used for all the atoms and for the metal center, respectively [68]. The one electron term RI-SOMF(1X) was included in order to accelerate the SOC calculation.

The mechanistic study was carried out in a water environment at the B3LYP level, currently used to describe photoprocesses [55,69,70]. The identity of the optimized structures as minima and transition states was checked through a normal mode vibrational analysis. These calculations were also used for obtaining the free energy values. An IRC algorithm was used to check the proper connection of the transition states with the corresponding minima.

## 4. Conclusions

A comprehensive computational exploration of the mechanism of action of a recently synthesized Ru(II) complex was carried out. The complex, proposed as a PACT compound, bears the chemotherapeutic drug sorafenib as a photo-labile ligand. DFT and time-dependent approaches were used to describe the photophysical properties, which include the characterization of each electronic transition as well as of the excited states potentially involved in the light-promoted generation of singlet oxygen and the active di-aquo complex along with the computation of the kinetic constant for ISC from the lowest-lying singlet state to triplet manifold. The whole pathway, which, under light irradiation, leads to the ligand release accompanied by the formation of the corresponding diaquo complex, able to bind to DNA, inducing cell death, has been described. In particular, it goes through three essential steps: (i) irradiation that entails a metal-to-ligand charge transfer state ^1^MLCT to be populated; (ii) a radiationless ISC transition from the ^1^MLCT state to the closest lying triplet ^3^MLCT_3_ state, followed by internal conversion to populate the ^3^MLCT_1_ state, from which the overcoming of a very low energy barrier leads to the formation of the lowest-lying triplet state ^3^MC, the pivotal excited state in the photoactivation reaction; followed by (iii) the formation of the diaquo complex able to bind DNA. The last step of the active species formation requires a multi-stages reaction according to which the primary removal of the chloride from the coordination sphere of the metal affords a penta-coordinated complex intermediate. The incoming water molecule, instead, entails the release of the sora ligand. Therefore, the excitation and hence the triplet state population is essential for the release of both the leaving ligands, chloride, and sora. From here, the complex needs to decay to the ground state in order for the second water molecule entrance to occur, which is accomplished by overcoming a very low energy barrier. The subsequent binding of the formed complex, Ru-2H_2_O, to DNA, here simulated with guanine nucleobase, requires an energy cost of no more than 10.4 kcal mol^−1^.

The outcomes reported here show that the proposed Ru(II) complex is able to efficiently generate ^1^O_2_, release the chemotherapeutic agent sorafenib thanks to a triplet state with an MC character with suitable energy and, at the same time, exert a chemotherapeutic action by preventing the replication and transcription of DNA. The computational investigation of the photoactivation process of the Ru(II)-based complex allowed us to describe in detail the steps leading to the formation of the active aqueous species, responsible for DNA distortion, and could help in the designing of efficacious Ru complexes suitable for PACT application. 

## Figures and Tables

**Table 1:** VEA and VIP values computed in water for ^3^O_2_ and **RuS** complex in its ground ([Ru]^+^) and triplet excited (^3^[Ru]^+^) states computed at the PBE0/6-31G(d) level.

	[Ru]^+^	^3^[Ru]^+^	O_2_
VEA ^a^	−2.49	−4.52	−2.09
VIP ^a^	5.17	3.13	-

^a^. vertical electron affinity (VEA) and ionization potential (VIP) are reported in (eV).

## Data Availability

The raw data supporting the conclusions of this article will be made available by the authors on request.

## Supplementary Materials

The following supporting information can be downloaded at: https://www.mdpi.com/article/10.3390/molecules29184298/s1, Table S1: Benchmark of exchange and correlation functional λ_max_ of Ru-Sora complex. Table S2: TDA-DFT outcomes for the investigated Ru complexes, Ru-Sora, RuS and Ru. Figure S1: The decomposition analysis for the low-lying triplet states T1–T8. Table S3: Energy splitting ΔE (eV) between the lowest singlet state S1 and triplet states lying below at their equilibrium geometry. Spin-orbit coupling elements SOC (cm^−1^) calculated at each triplet-state optimized structure. Kinetic constant k_ISC_ (s^−1^) for ISC. Figure S2: Relaxed potential energy scan from the ^3^Ru_MLCT_ state to ^3^Ru_MC_. Figure S3: Optimized structures of all the stationary points intercepted along the free energy profile of Figure 6 of the manuscript. Figure S4: Optimized structures of the minima intercepted along the pathway for guanine coordination.
