# Supplementary material for: Computational Exploration of the Mechanism of Action of a Sorafenib-Containing Ruthenium Complex as an Anticancer Agent for Photoactivated Chemotherapy
Source: Molecules. 2024 Sep 11;29(18):4298. doi: 10.3390/molecules29184298 (PMC11433670; doi:10.3390/molecules29184298)
Supplement: Supplementary file 1 [file molecules-29-04298-s001.zip › molecules-3150017-supplementary.pdf]

Supplementary Materials

Computational Exploration of the Mechanism of Action of a Sorafenib-Containing Ruthenium Complex as an Anticancer Agent for Photoactivated Chemotherapy

Pierraffaele Barretta [1], Fortuna Ponte [1,*], Daniel Escudero [2] and Gloria Mazzone [1,*]

[1] Department of Chemistry and Chemical Technologies, University of Calabria, Via P. Bucci, 87036 Rende (CS), Italy; pierraffaele.barretta@unical.it
[2] Department of Chemistry, KU Leuven, Celestijnenlaan 200F, 3001 Heverlee, Belgium; daniel.escudero@kuleuven.be
* Correspondence: fortuna.ponte@unical.it (F.P.); gloria.mazzone@unical.it (G.M.)

Table of Contents

- **Table S1**. Benchmark of exchange and correlation functional λ_{max} of **Ru-Sora** complex. S2
- **Table S2.** TDA-DFT outcomes for the investigated Ru complexes, **Ru-Sora, RuS** and **Ru**. S3
- **Figure S1**. The decomposition analysis for the low-lying triplet states T1-T8 S4
- **Table S3**. Energy splitting ΔE (eV) between the lowest singlet state S1 and triplet states lying below at their equilibrium geometry. Spin-orbit coupling elements SOC (cm^{-1}) calculated at each triplet state optimized structure. Kinetic constant k_{ISC} (s^{-1}) for ISC. S5
- **Figure S2.** Relaxed potential energy scan from the $^3Ru_{MLCT}$ state to $^3Ru_{MC}$. S6
- **Figure S3**. Optimized structures of all the stationary points intercepted along the free energy profile of Figure 6 of the manuscript. S7
- **Figure S4**: Optimized structures of the minima intercepted along the pathway for guanine coordination S8

Table S1. Benchmark of exchange and correlation functional λ_{max} of **Ru-Sora** complex

	**λ_{max} (nm)**	**Δ**
**exp**	465	
**B3LYP**	544.0	79.0
**CAM-B3LYP**	417.0	-48.0
**B3PW91**	548.0	83.0
**M06**	563.0	98.0
**M06L**	672.5	207.5
**PBE**	748.9	283.9
**PBE0**	**511.0**	46.0
**LC-WHPBE**	393.4	-71.6
**ωB97**	397.2	-67.8
**B97D**	736.0	271.0
**ωB97X**	399.9	-65.1
**ωB97XD**	425.0	-40.0

Table S2. TDA-DFT outcomes for the investigated Ru complexes, **Ru-Sora, RuS** and **Ru.**

Band	ΔE	λ	*f*	MO Contribution	Theoretical Assignment
				**Ru-Sora**	
I	2.43	511	0.004	H → L 70%; H → L+1 20%	MLCT/LLCT
	2.79(S5)	444	0.120	H-2 → L 40%; H-1 → L 32%; H-1 → L+1 10%	MLCT/ILCT
	2.97	416	0.058	H-2 → L+1 50%; H → L+1 16%; H-1 → L 11%	MLCT/ILCT
II	3.71	335	0.055	H → L+5 52%; H-1 → L+4 14%	ML_1CT/MLCT
	3.79	327	0.074	H-2 → L+3 34%; H-1 → L+4 32%	ML_1CT/MLCT
	3.93	316	0.044	H-2 → L+4 61%; H-2 → L+5 14%	MLCT/IL_1CT
				**RuS**	
I	2.42	513	0.004	H → L 69%; H → L+1 21%	MLCT/LLCT
	2.78	445	0.124	H-2 → L 45%; H-1 → L 34%	MLCT/ILCT
	2.97	418	0.054	H-2 → L+1 47%; H+1 → L 16%; H-1 → L 12%	MLCT/ILCT
II	3.71	334	0.044	H → L+5 41%; H-1 → L+4 21%; H-1 → L+3 12%	ML_1CT/MLCT
	3.81	326	0.068	H-1 → L+5 45%; H → L+5 12%; H-2 → L+3 11%; H → L+6 10%	ML_1CT/MLCT
	3.83	324	0.051	H → L+6 50%; H-1 → L+5 25%	MLCT
				**Ru**	
I	2.43	511	0.004	H → L 70%; H → L+1 20%	MLCT/LLCT
	2.79	444	0.120	H-2 → L 40%; H-1 → L 32%; H-1 → L+1 10%	MLCT/ILCT
	2.97	417	0.058	H-2 → L+1 50%; H → L+1 16%; H-1 → L 11%	MLCT/ILCT
II	3.71	335	0.055	H → L+5 52%; H-1 → L+4 14%	ML_1CT/MLCT
	3.79	327	0.074	H-2 → L+3 34%; H-1 → L+4 32%	ML_1CT/MLCT
	3.93	316	0.044	H-2 → L+4 61%; H-2 → L+5 14%	MLCT/IL_1CT

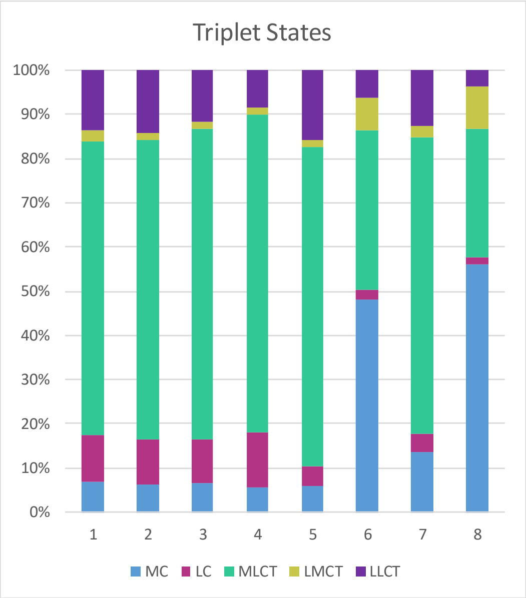

Figure S1. The decomposition analysis for the low-lying triplet states T1-T8.

Table S3. Energy splitting ΔE (eV) between the lowest singlet state S_1 and triplet states lying below at their equilibrium geometry. Spin-orbit coupling elements SOC (cm^{-1}) calculated at each triplet state optimized structure. Kinetic constant k_{ISC} (s^{-1}) for ISC.

	**S_1-^{3}MC**	**S1-^{3}MLCT$_1$**	**S1-^{3}MLCT$_2$**	**S1-^{3}MLCT$_3$**
ΔE (eV)	0.39	0.19	0.06	0.01
SOC (cm^{-1})	50.7	135.3	87.3	278.5
k_{isc} (s^{-1})	$4.53 \cdot 10^{-1}$	$6.92 \cdot 10^{9}$	$6.39 \cdot 10^{8}$	$1.24 \cdot 10^{11}$

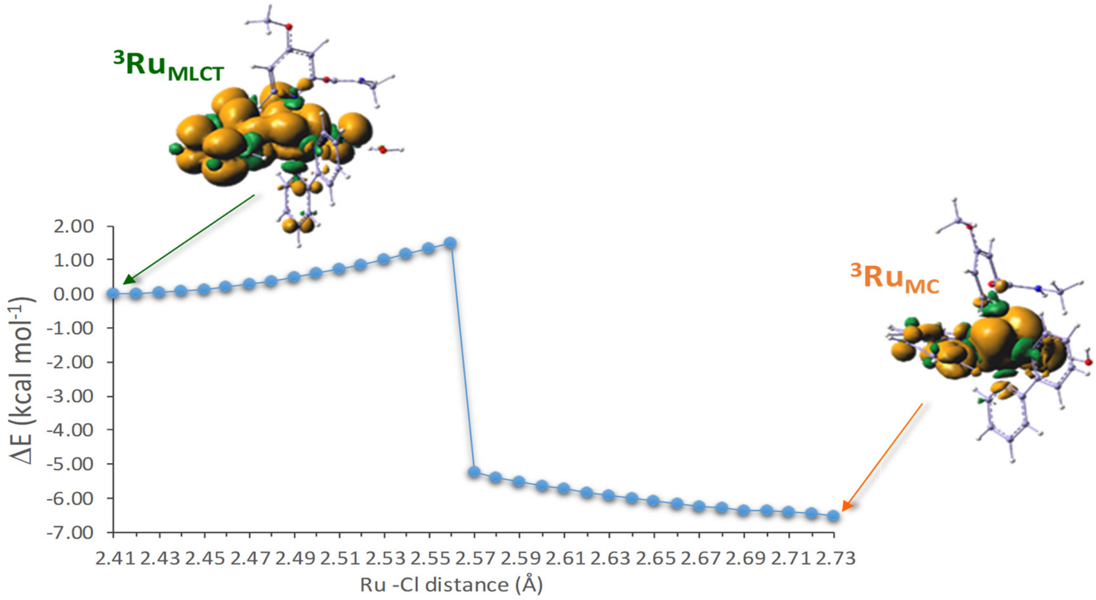

Figure S2. Relaxed potential energy scan from the $^3Ru_{MLCT}$ state to $^3Ru_{MC}$. Spin density plots of the two states are also provided.

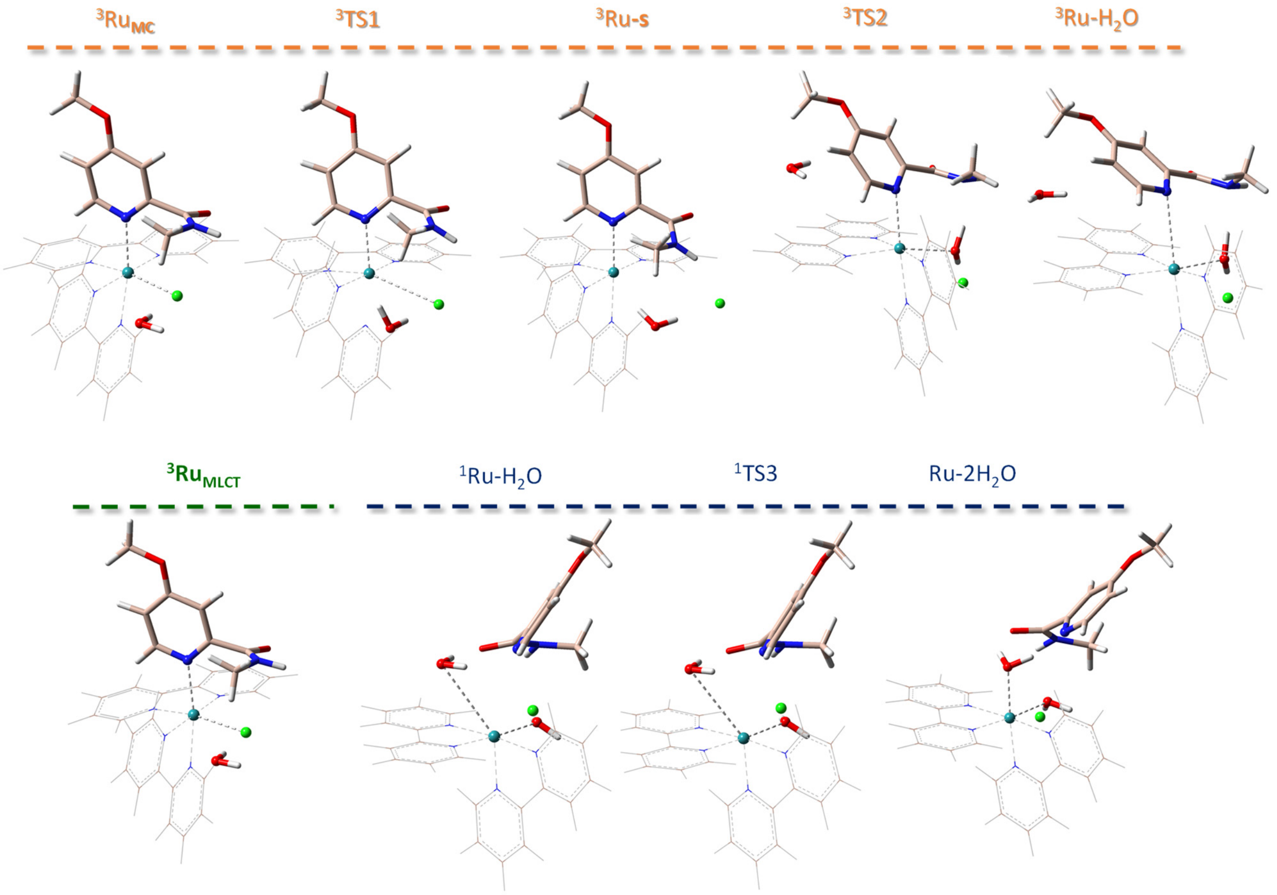

Figure S3. Optimized structures of all the stationary points intercepted along the free energy profile of Figure 6 of the manuscript.

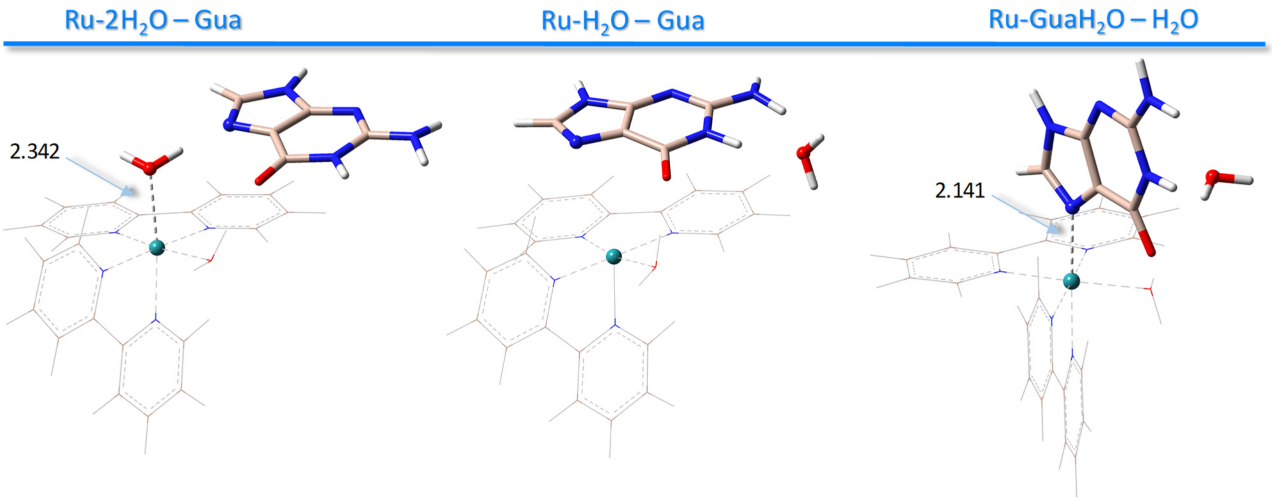

Figure S4: Optimized structures of the minima intercepted along the pathway for guanine coordination.
